# Transthoracic echocardiography and its limitations in the diagnosis of congenital supernumerary aortic valve in a Thoroughbred

**DOI:** 10.1002/vms3.472

**Published:** 2021-03-13

**Authors:** Valentina Vitale, Malene Laurberg, Gaby van Galen

**Affiliations:** ^1^ University Teaching Hospital Sydney School of Veterinary Science University of Sydney Camden NSW Australia; ^2^ Present address: Veterinary Teaching Hospital “Mario Modenato”, Department of Veterinary Sciences University of Pisa Pisa Italy

**Keywords:** aortic malformation, congenital abnormality, equine, quadricuspid aortic valve, valvulopathy

## Abstract

Aortic valve malformation is a common congenital abnormality reported in human medicine. The malformation is characterised by an increased or decreased number of cusps. Anatomical variations of the aortic valve that have been documented in humans include unicuspid, bicuspid, quadricuspid and quinticuspid valves. Two reports described a quadricuspid aortic valve in horses associated with either a ventricular septal defect (VSD) or tetralogy of Fallot. In this case report we describe the clinical and echocardiographic findings of a horse with a quadricuspid aortic valve as single congenital abnormality, referred with history of exercise intolerance and an episode of paroxysmal atrial fibrillation. Limitations and risks of misdiagnosis that can be encountered with transthoracic echocardiography are also discussed. The reported case highlights the importance of echocardiographic screening in asymptomatic patients as congenital heart disease can be present without obvious cardiac signs. As advanced imaging on the equine thorax is still far from future possibilities for adult horses, this report may help to reach an accurate diagnosis with similar cases.

## Graphical abstract

In this case report we describe the clinical and echocardiographic findings of a horse with a quadricuspid aortic valve as single congenital abnormality. Limitations and risks of misdiagnosis that can be encountered with transthoracic echocardiography are discussed. The reported case highlights the importance of echocardiographic screening in asymptomatic patients as congenital heart disease can be present without obvious cardiac signs.

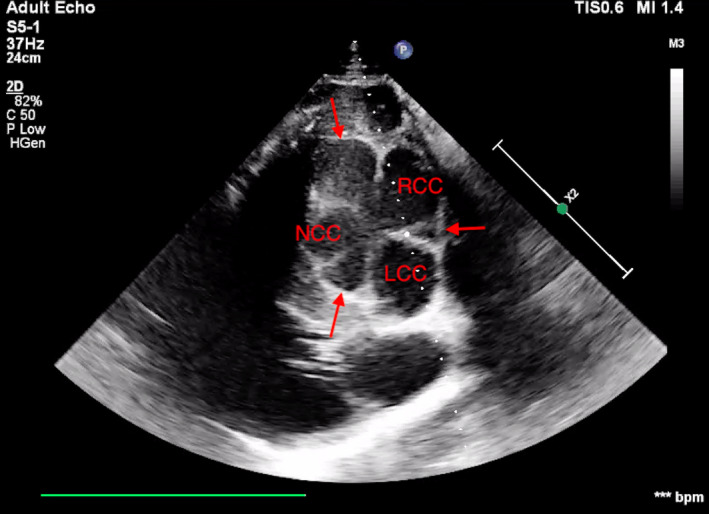

## 1 INTRODUCTION

Aortic valve malformation is a common congenital abnormality reported in human medicine (Meng et al., 2009). The malformation is characterised by an increased or decreased number of cusps. Anatomical variations of the aortic valve that have been documented in humans include unicuspid, bicuspid, quadricuspid and quinticuspid valves (Meng et al., 2009; Oladiran et al., 2019). Of these variants the bicuspid valve (BAV) is the most common with a reported prevalence of ~1% with male predominance (Meng et al., 2009; Sharif et al., 2019; Sun et al., 2019). Prevalence of quadricuspid aortic valve (QAV) is significantly lower (0.06%), and only three sporadic reports exist in human literature that documented a quinticuspid aortic valve (Bogers et al., 1982; Meng et al., 2009; Simonds, 1923).

There are few reports of BAV in the dog (Scansen et al., 2018; Winter et al., 2019), in a sea lion (Koutaka et al., 2017) and in donkeys (Lilleengen, 1934; Rooney & Franks, 1964). QAV has been diagnosed in dogs (Serres et al., 2008), in hamsters (Fernandez et al., 1999; Lopez‐Garcia, 2015) in a shrew (Duran, 1996), in a cat (Nakamura et al., 2015) and in a horse (Michlik et al., 2014).

Diagnosis of these congenital malformations is usually reached with transthoracic echocardiography, but in humans advanced diagnostic imaging, such as transesophageal echocardiography (TEE), magnetic resonance imaging and computerised tomography, provides a more accurate assessment of valvular anatomy (Meng et al., 2009; Oladiran et al., 2019). In dogs, TEE is routinely used to better characterise the type of abnormality (Winter et al., 2019). However, in some cases, the diagnosis is reached as an incidental finding at necropsy (Koutaka et al., 2017; Oladiran et al., 2019).

Aortic valve malformations usually present as isolated congenital anomalies but have also been reported associated with other cardiac abnormalities, including aortic root dilatation, tetralogy of Fallot, patent ductus arteriosus, atrial and ventricular septal defects (VSDs) and anomalous origin of the coronary arteries (Oladiran et al., 2019).

In the two cases described in horses the quadricuspid aortic valve was associated with a VSD and consequent cardiomegaly in one horse (Michlik et al., 2014) and with tetralogy of Fallot in the other (Gesell & Brandes, 2006).

The purpose of the present case report is to describe the clinical and echocardiographic findings in a horse presenting a quadricuspid aortic valve as single congenital abnormality, including the limitations and risks of misdiagnosis that can be encountered with transthoracic echocardiography.

## 2 CASE REPORT

### 2.1 Case history

A 4‐year‐old Thoroughbred gelding, weighing 502 kg, was referred to the Camden Equine Centre of the Veterinary Teaching Hospital of the University of Sydney with history of poor performance on a race (one week before admission) and during training. Post racing examination and electrocardiogram recording performed immediately post racing revealed atrial fibrillation (AF). The horse had, however, reconverted spontaneously to sinus rhythm few hours later. The trainer mentioned that during light exercise the horse performs well, but during the last 6 months the horse repeatedly stopped when pushed to high‐speed gallop without additional clinical signs.

### 2.2 Clinical findings

**FIGURE 1:**
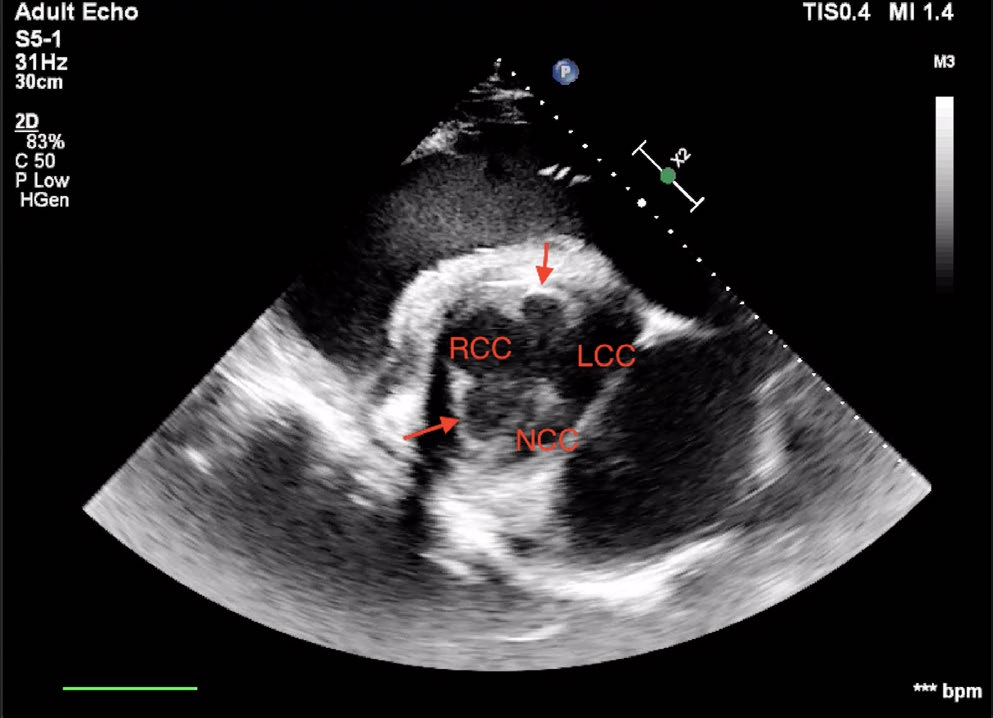
B‐mode right parasternal short‐axis left ventricular outflow tract view at the level of Valsalva's sinuses. RCC, right coronary cusp; LCC, left coronary cusp; NCC, noncoronary cusp. Arrows point the additional cusps observed

**FIGURE 2:**
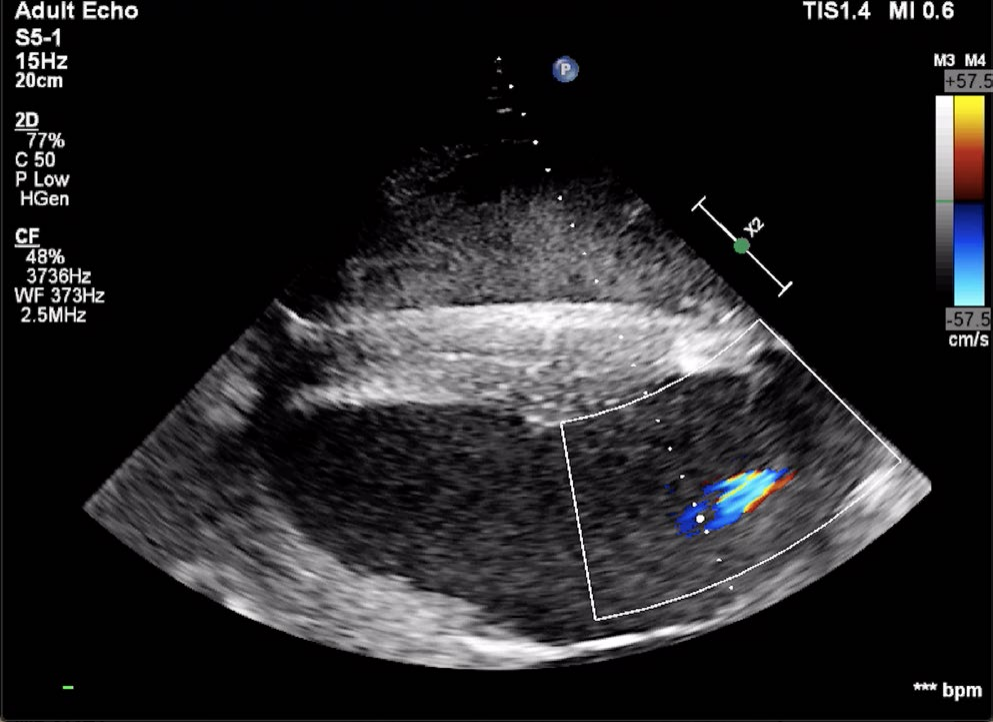
Transthoracic color Doppler echocardiography in the right parasternal long‐axis left ventricular outflow tract view shows mild aortic regurgitation

**FIGURE 3:**
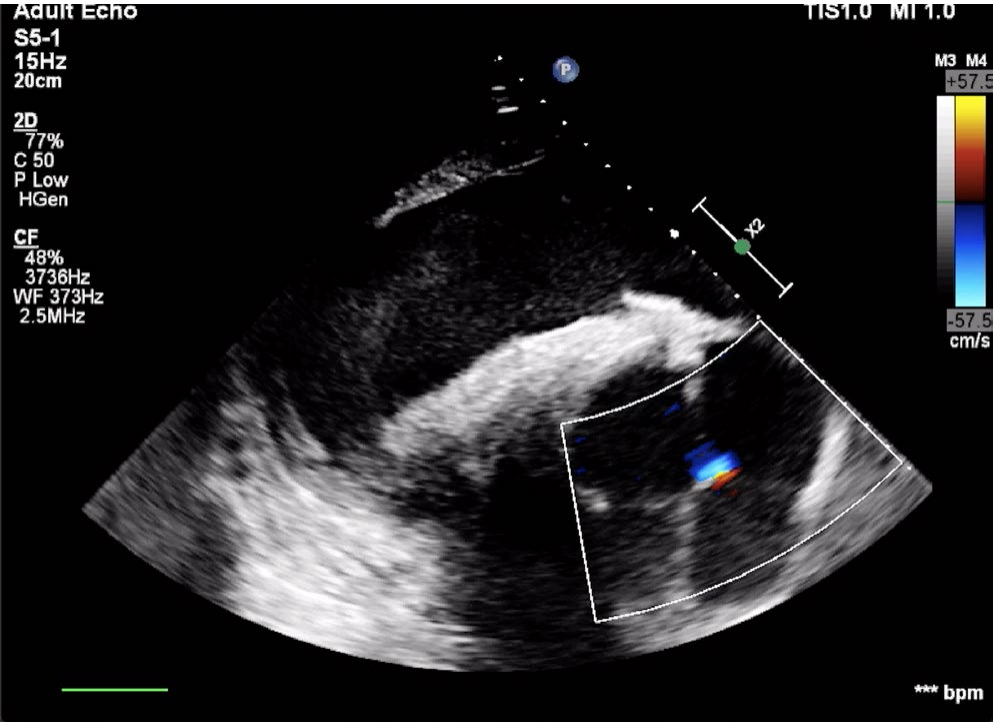
Transthoracic color Doppler echocardiography in the right parasternal short‐axis left ventricular outflow tract view

**FIGURE 4:**
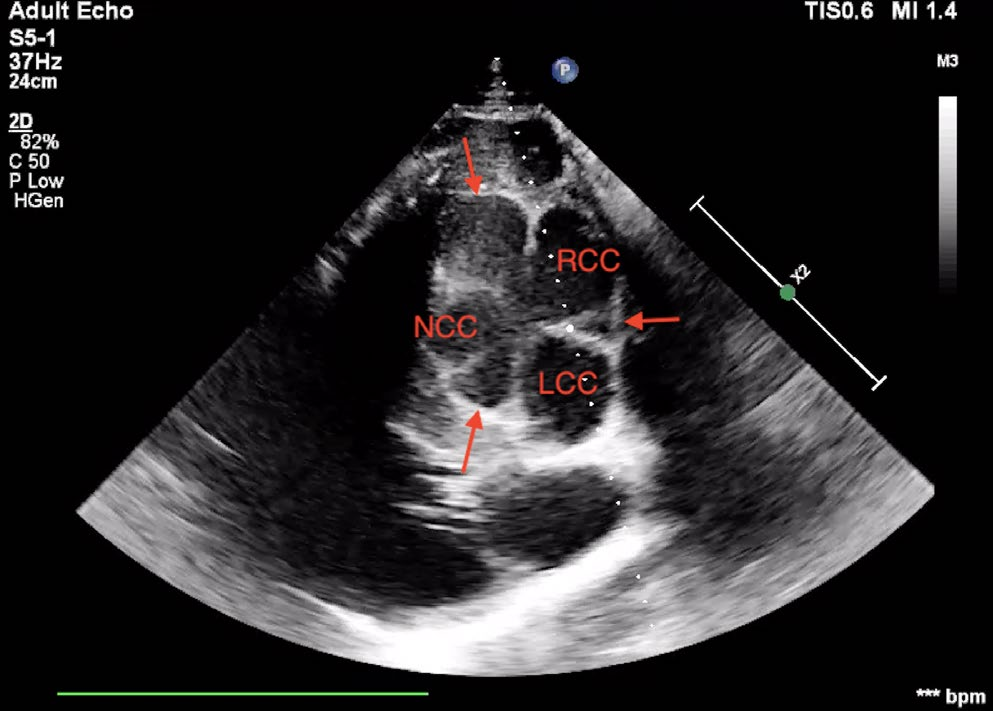
B‐mode left parasternal short‐axis left ventricular outflow tract view at the level of Valsalva's sinuses. RCC, right coronary cusp; LCC, left coronary cusp; NCC, noncoronary cusp. Arrows point the additional cusps observed

Physical examination on arrival and blood analysis, including cardiac troponin I, was unremarkable. Cardiac auscultation with conventional stethoscope (3M^TM^ Littmann® Cardiology IV^TM^ Stethoscope) did not reveal any murmur or arrhythmia. A transthoracic echocardiographic examination was carried out according to a previously published protocol (Marr & Patteson, 2010) using an ultrasound system (Philips EPQ 5G, Release 1.5.2) with a 2.5 MHz phased‐array transducer with harmonic imaging. From the B‐mode right parasternal short‐axis left ventricular outflow tract view, two supernumerary aortic cusps were suspected, one on each side of the right coronary cusp (Figure 1). Transthoracic color Doppler echocardiography in the right parasternal long‐axis on left and right ventricular outflow tract views permitted to identify aortic (Figure 2) and pulmonary regurgitation. The aortic regurgitation (AR) was visible with color Doppler also on the short‐axis view (Figure 3). Based on a previously described scoring system (Ven et al., 2016), AR was considered mild. The regurgitant jet was small and only visible in a very specific location on the long‐axis view; thus, it was not easy to obtain an adequate image with the spectral Doppler. Due to the fact that it would most likely be encountered with an error, we decided to forfeit this measurement. Similarly, the pulmonic regurgitation was considered mild, based on the limited extension of the regurgitant jet (<1/3 of right ventricle). No mitral nor tricuspid regurgitation were observed. Furthermore, from the B‐mode left parasternal short‐axis left ventricular outflow tract view, two supernumerary aortic cusps were also observed but this time they seemed to be on each side of the noncoronary cusp, with a possible additional small cusp between right and left coronary cusps (Figure 4).

The evaluation of cardiac structures was performed using B‐ and M‐mode echocardiography. The results of the M‐mode measurements are presented in Table 1. The normal echocardiographic measurements are based on previously reported values (Patteson et al., 1995).

**TABLE 1:** Measured cardiac dimensions and normal echocardiographic M‐mode values in horses (Patteson et al., 1995)

Parameter	Patient	Reference range
IVSd (cm)	3.03	2.73 ± 0.354
IVSs (cm)	4.68	4.21 ± 0.347
LVIDd (cm)	10.7	10.67 ± 0.748
LVIDs (cm)	6.86	6.44 ± 0.861
FS (%)	41.7	39.87 ± 4.83

Note

Abbreviations: FS, shortening fraction; IVSd, interventricular septum thickness in diastole; IVSs, interventricular septum thickness in systole; LVIDd, left ventricular internal diameter in diastole; LVIDs, left ventricular internal diameter in systole.

A base‐apex standard electrocardiographic examination was then performed at rest, during and after exercise on treadmill using a telemetry system (Televet 100 ECG & Holter). A heart rate of 200 bpm was reached and maintained for approximately 60 s on treadmill at fast gallop (9 m/s). No arrhythmias were observed, and recovery was excellent with a heart rate of 80 bpm within 5 min post‐exercise and below 60 bpm within 10 min.

### 2.3 Diagnosis

Based on echocardiographic finding,s a diagnosis of aortic valve dysplasia with associated mild regurgitation was made. Combining the images from right and left parasternal short axis views, one could have suspected the presence of six aortic cusps. Nevertheless, our opinion is that there was only one extra leaflet that was consistently visible; thus, the abnormality could be classified as four equal cusps according to the literature (Hurwitz & Roberts, 1973). This leads to the conclusion that the horse had one right coronary cusp, 1one left coronary cusp and two noncoronary cusps. The presence of an extra noncoronary cusp possibly caused all the cusps to be more concave than usual. Transsectioning them with ultrasonography at different moments during their opening and closing movement can then lead to the inconsistent effect of the presence of even more leaflets.

### 2.4 Outcome

Based on the findings and the history of AF, although AR was mild and unlikely to affect performance, the owner preferred to retire the horse from the athletic career.

## 3 DISCUSSION

Aortic valve abnormalities have been well documented in human medicine (Oladiran et al., 2019). The superior engineering properties of the tricuspid aortic valve were firstly recognised by Leonardo da Vinci (Da, 2002). Indeed, human patients with BAV or QAV frequently present aortic insufficiency or stenosis (Oladiran et al., 2019). The cause of the occurrence of supernumerary cusps is not completely understood but it is believed to result from aberrant division of one of the three mesenchymal ridges that normally gives rise to three aortic valve cushions (Fernandez et al., 1999; Oladiran et al., 2019). Although the embryological development is a continuous process, three critical phases with regard to the supernumerary‐cusped aortic valve can be recognised. Firstly, supernumerary prevalvular pads in the embryological truncus arteriosus can evolve in supernumerary‐cusped aortic or pulmonary valves after separation into aorta and pulmonary artery. Secondly, a normal number of prevalvular pads in the truncus arteriosus can become excessively divided because of an abnormal dividing pattern of aorta and pulmonary artery, thereby producing a supernumerary cusped aortic or pulmonic valve. Or, in last instance, after normal separation of the aorta and pulmonary artery, the prevalvular pads can develop in an abnormal way to form a supernumerary‐cusped valve (Bogers et al., 1982). Based on that, if the pulmonary valve is normal, the congenital abnormality likely developed after the separation of aorta and pulmonary artery. Unfortunately, defining the morphology of the pulmonary artery is difficult and limited with standard transthoracic echocardiography and many alterations can remain undetected (Bogers et al., 1982; Oladiran et al., 2019). The described horse also had a regurgitation on this valve. And therefore, even though mild pulmonary regurgitation constitutes a common finding in horses (Blissitt, 2010), the possibility to a concomitant anomalous pulmonary valve cannot be entirely ruled out in this horse.

Echocardiography utilises the physical properties of ultrasound waves to construct images of cardiac tissue and structures. Ultrasound waves travelling through biological tissue typically obey the laws of reflection and refraction (Bertrand et al., 2016). Thus, when dealing with concave/convex structures in rapid movement, such as the cardiac valves, it becomes difficult to interpret the images obtained on the screen. The presence of four sinuses of Valsalva, as documented on both Figures 1 and 4, confirms the presence of four leaflets. However, it also rules out the presence of more than four, as the lack of consistent presence of more than four cusps in multiple views (Bertrand et al., 2016) is in favour of an artefact than of a real structure. To differentiate between artefacts and real structures, logical anatomical relationship should be identified (Bertrand et al., 2016). Comparing the views from right and left sides of the thorax permitted to confirm the presence of two noncoronary cusps between the left and right coronary cusps but showed inconsistency about the other possible additional leaflets, making their real presence less likely.

The use of TEE could have helped in confirming the exact number of supernumerary cusps (Meng et al., 2009; Oladiran et al., 2019; Winter et al., 2019), but this is not commonly available in equine practise and also not at our facility. A 3D echocardiography has been described in human (Shiota, 2008) and veterinary medicine (Menciotti et al., 2018) and has been applied also in equine cardiology (Redpath et al., 2020; Worsman et al., 2020). This technique provides greater spatial recognition of structures in the heart and has been successfully used to better characterise an atrial septal defect in a horse (Redpath et al., 2020). The improved imaging quality of 3D echocardiography could have helped also in our case to identify the exact location and size of the supernumerary aortic cusp. The absence of a murmur in our patient is not surprising as the loudness of an aortic murmur on auscultation does not necessarily relate to the severity of the regurgitation (Keen, 2016). Also in other species, despite a documented AR on ultrasound, no murmur was auscultated (Meng et al., 2009; Nakamura et al., 2015). Furthermore, aortic malformations have been frequently reported asymptomatic in human medicine (Oladiran et al., 2019). This highlights the importance of echocardiographic screening in asymptomatic patients (Nakamura et al., 2015); a horse such the described patient would have easily passed a prepurchase examination despite the presence of a congenital aortic malformation with associated valvular regurgitation. Valves with abnormal numbers of cusps may open improperly causing stenosis or may close incompletely resulting in regurgitation. Patients with these abnormalities may expect to develop hemodynamically significant complications at some point in their lifetime (Meng et al., 2009); thus, close follow‐up is recommended (Oladiran et al., 2019). For symptomatic patients, in human medicine valve repair and valve replacement have been described and this can be applied also in dogs (Winter et al., 2019). In horses this has not been described yet.

Although in horses AR is unlikely to affect performance, unless it is severe, the risks of AF, pulmonary hypertension and congestive heart failure are increased (Reef et al., 2014). In humans, AF has been reported in association of quadricuspid aortic valve and its cause has been attributed to left ventricular hypertrophy based on voltage criteria (Oladiran et al., 2019). In our patient the reported past episode of AF that spontaneously reconverted could have been the first sign of increased arrhythmogenic risk.

## 4 CONCLUSIONS

We reported here a case of quadricuspid aortic valve as single congenital abnormality in a 4‐year‐old Thoroughbred gelding and detailed the difficulties and risk of misdiagnosis that a transthoracic echocardiography includes, in the hope that our report can help with similar cases to reach an accurate diagnosis, as advanced imaging on the equine thorax is still far from future possibilities for adult horses.

## CONFLICT OF INTEREST

The authors have no conflicting interest to declare.

## AUTHOR CONTRIBUTION

**Valentina Vitale:** Investigation; Writing‐original draft. **Malene Laurberg:** Investigation; Methodology. **Gaby Van Galen:** Conceptualization; Writing‐review & editing.

### PEER REVIEW

The peer review history for this article is available at https://publons.com/publon/10.1002/vms3.472.
